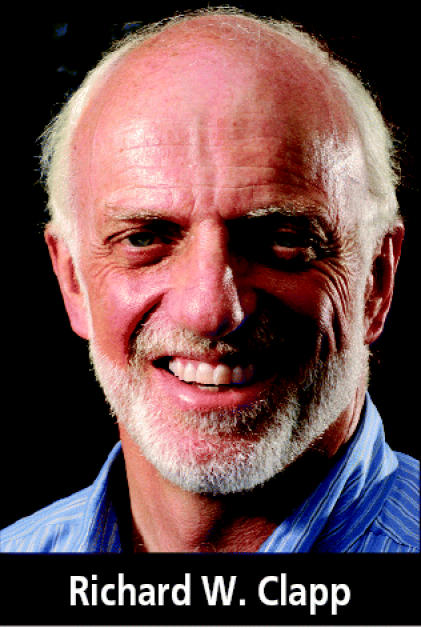# Guest Editorial: Nuclear Power and Public Health

**DOI:** 10.1289/ehp.113-a720

**Published:** 2005-11

**Authors:** Richard W. Clapp

**Affiliations:** Department of Environmental, Health Boston University School of Public Health Boston, Massachusetts E-mail: rclapp@bu.edu

Because of concern about the health and environmental effects of burning fossil fuels such as coal and oil to produce electrical energy in recent years, there has been a resurgence of interest in nuclear power stations as a “carbon-free” method of generating electricity. For example, an interdisciplinary study titled The Future of Nuclear Energy, released by the Massachusetts Institute of Technology (MIT 2003) suggests that there are four options for reducing carbon dioxide emissions from electricity: increasing efficiency, expanding renewable energy sources, capturing carbon dioxide and sequestering the carbon, and increasing use of nuclear power. This study and one of its authors have gotten considerable public attention in the past couple of years for putting the nuclear power option back on the table for discussion in the United States (Baue 2005).

Furthermore, evolving nuclear power plant technologies, including one design that has been described as inherently safe (Uranium Information Centre 2005), have been making their way through the review and approval processes in other countries. The so-called pebble bed modular reactor (PBMR) is now being proposed for construction in Cape Town, South Africa, and there is a lively debate in that country about whether such a design is verifiably safe, whether the country truly needs such a power plant, where the waste would be sent and whether those affected would have a political voice in the debate, and ultimately how the effort to approve and construct a PBMR in South Africa would impact this industry in the rest of the world (Groenewald 2005; Nuclear Engineering Department, MIT 2001). This debate is still under way, and it is being watched closely by interested parties. Updated plans for the South African project will be submitted to the utility regulatory body in 2006. The PBMR design would not be acceptable under current U.S. regulations because it does not require an expensive containment dome; this means that it is less expensive to build but is more vulnerable from a security standpoint.

A further question about the security of existing U.S. nuclear power plants arose in the aftermath of the 11 September 2001 attacks on the World Trade Center and the Pentagon. Nuclear power plants appear to have been a potential target of the organizers of these attacks; therefore, the exposure of spent fuel in aboveground storage tanks on the property of many commercial plants is a concern. New security arrangements and proposals for more secure dry-storage casks have been made, but long-term secure storage of high-level reactor waste remains an unresolved problem.

As the discussion of the nuclear power option moves forward, it is critically important to consider what is now known about the health and environmental risks of the nuclear fuel cycle, based on the lessons of the past 60 years (Cardis et al. 2005; Wing et al. 1997). There is now a large body of knowledge about the impact of uranium mining and milling, transportation of partially enriched ore, fabrication of fuel-grade material, power reactor operations, and waste disposal and decommissioning of commercial reactors. We have learned from disasters such as Chernobyl [United Nations Scientific Committee on the Effects of Atomic Radiation (UNSCEAR) 2000], as well as the less obvious but long-term problems of disposal of mine wastes and mill tailings and the ecologic impacts of this technology (Makhijani et al. 1996). We have also learned about the human health effects of low-level radiation exposure on workers exposed in the nuclear industry, most recently summarized in the BEIR (Biological Effects of Ionizing Radiation) VII report [National Academy of Sciences (NAS) 2005]. The conclusions of this recent review, although couched in careful scientific language, indicate that carcinogenic effects of exposure increase proportionately with dose, especially regarding leukemia mortality, and that for some types of exposure the current regulatory controls in the United States may be insufficient. A proliferation of nuclear power plants inevitably means more nuclear workers and more residents exposed to low-level ionizing radiation, with increased health risks attendant to this exposure.

*The Future of Nuclear Energy* (MIT 2003) suggests that nuclear power generation of electricity is currently not cost-effective compared with other technologies. The report notes that “carbon emission credits, if enacted by government, can give nuclear power a cost advantage” (MIT 2003). In fact, there are several other carbon-free or low-carbon options that are currently more cost-effective than nuclear power; these include wind power, combined-cycle gas power plants, and end-use efficiency measures. According to a recent analysis, “nuclear power saves as little as half as much carbon per dollar as windpower and cogeneration, and from several-fold to at least tenfold less carbon per dollar as end-use efficiency” (Lovins 2005). Lovins (2005) also succinctly added that

No other energy technology spreads do-it-yourself kits and innocent disguises for making weapons of mass destruction, nor creates terrorist targets or potential for mishaps that can devastate a region, nor creates wastes so hazardous, nor is unable to restart for days after an unexpected shutdown.

The accumulated experience of the past six decades provides ample evidence of adverse health effects in workers in the nuclear fuel cycle, the potential for disastrous accidents that lead to widespread environmental contamination, the unresolved problems of permanent and secure storage of high-level radioactive wastes, and the extraordinarily high costs of building additional nuclear power generation facilities. Some of these problems are ignored in the current public discourse, perhaps because of the immediacy of the need to solve the problems of carbon-based fuel. Given the availability of alternative carbon-free and low-carbon options and the potential to develop more efficient renewable technologies, it seems evident that public health would be better served in the long term by these alternatives than by increasing the number of nuclear power plants in the United States and the rest of the world.

## Figures and Tables

**Figure:**